# Sports Spectating in Connected Stadiums: Mobile Application Roland Garros 2018

**DOI:** 10.3389/fspor.2022.802852

**Published:** 2022-03-18

**Authors:** Pierre-Olaf Schut, Ekaterina Glebova

**Affiliations:** ^1^Université Gustave Eiffel, Analyse Comparée des Pouvoirs (ACP) - EA 3350, Champs-sur-Marne, France; ^2^Université Paris-Saclay CIAMS, Orsay, France; ^3^Université d'Orléans, CIAMS, Orléans, France

**Keywords:** mobile application, sport, tennis, user experience, internet, connected stadium, customer experience, Roland Garros

## Introduction

The mobile applications (apps) industry is evolving and uptake all possible fields of life and activity, for example, reservations, gaming, gambling, paying bills, ordering tickets, communications, broadcastings, search, and leisure time. Consumers' use of mobile applications brings multiple challenges for research in consumer behavior (Buck et al., 2014; Kang et al., 2015; Hwang et al., 2020) and businesses have seen this type of technology (Glebova and Desbordes, 2021) as a new way to communicate with customers (Vikström and Zheng, 2013; Næss, 2016; Glebova et al., 2019). The sports industry has been benefiting from this evolution (Greenhalgh et al., 2014; Glebova and Desfontaine, 2020). Mobile apps became an essential part of sports culture, functioning for all the stakeholders, giving a next-level fan experience (Glebova et al., 2019). Recent academic research demonstrates the role of different types of media in sports fans' perceptions and experiences in the stadium (Behrens and Uhrich, 2021). Furthermore, stadium visitors integrate sport event-related and -unrelated digital resources, especially apps, to co-create value at sport events (Horbel et al., 2021).

This paper aims to overview the key points of the role of mobile applications in today's sports user experiences (Næss, 2016; Kemppainen, 2018) and test interrelations between variables related to use a smartphone (Hwang et al., 2020) on sporting events and apps' employment for sports consumers in the context of sports and a connected stadium, using an example of Roland Garros (RG) Mobile App 2018 (RGMA). Consequently, it extends literature and supports sport managers in an understanding of their audience as apps users, and, subsequently, helps to improve customer experience, using modern digital tools, in the RG stadium, and beyond.

### Case Context

One of the key advantages of mobile apps is the consolidation of all the services and data in a single “place.” Events, brands, organizations, and venues are developing their mobile app(s) because it is the “place” for all services and customer interactions. Access to all information and services is structured in a single system, easily accessible for a consumer (Glebova and Desfontaine, 2020). For example, a sports club may include their mobile stores as an online-commerce option into an app. It would help offer an inclusive and complex mobile experience for fans, allowing users to buy club memorabilia through their mobile devices. Relatively, the motto of the RGMA sounds like “All Roland Garros now in your smartphone.” It is an illustration of consolidating all the services and data at a single virtual place, a mobile application.

Roland Garros Mobile Application has been completely redesigned for the RG 2018 tournament, this redesign has led to a late (a couple of weeks before the tournament) release of the app on Apple and Google app stores. Therefore, users were unable to obtain the application in advance, before the start of the tournament. Accordingly, some RGMA functions were, probably, less successful than initially expected.

Roland Garros Mobile Application is a free-of-charge mobile application available on the Apple store and Google app store in versions for iOS and Android. It provides a set of media, news, information about all players (photo, name, ranking, date and place of birth, weight, height, main hand, and scores), matches scheduling with the sorting options, profile, notifications options (tournament alerts, matches, and calendar), shop (linked to store.rolandgarros.com), ticketing (linked to tickets.rolandgarros.com), help and support option, English or French language switch option, GDPR (audience tracking and advertising data), privacy policy. Overall, the set of RGMA tools and offered functions is quite standard and overlaps with plenty of similar today's mobile applications (Greenhalgh et al., 2014; Glebova and Desfontaine, 2020).

## Materials and Methods

### Research Focus

This study refers to the customer-centric approach, seeing visitors of RG as customers (Glebova and Desfontaine, 2020) through the prism of social constructivism (McKinley, 2015), where social factors contribute to the success of the failure of technology (in this case, it's RGMA and related connected facilities). According to social constructivism, society influences technology, in the opposition to technological determinism, where technology influences society (Winner, 2001; Wyatt, 2008). It brings to our attention the cultural lag theory as a difference between material culture (connected stadium RG and RGMA) and non-material culture (RGMA use, implementation, and employment; customers awareness and technology acceptance (Kang et al., 2015); and technology readiness). It explains that social (consumer) behavior takes time to catch up with technologies (Ogburn, 1922; Brinkman and Brinkman, 1997).

Our research incorporated two studies. Initially, using questionnaires, we sought to investigate how widespread was the use of RGMA 2018 and related connected facilities. Also, we are interested in outlining and understanding the key features of the consumption behavior of RGMA users.

We sought to answer exploratory and descriptive questions (Veal and Darcy, 2014):

1. What are the conditions for developing the connected stadium RG?
  1.1 What is the connected audience of RGMA?
  1.2 How is the audience affected by the app functions?
  1.3 What functions of RGMA have not been employed?
2. How do these digital tools influence consumer behavior?

The aim of the second study is to test, in more detail, specific differences in the consumption patterns of RGMA-users and non-users and develop a typology of RG visitors divided by dimension of approach to use RGMA. Both studies are conducted simultaneously, as the research progresses. Setting numbers (studies one and two) is used for the convenience of perception, but not to indicate the order of sequential actions.

### Data Collection

Initially, the questionnaire has been designed and conducted to explore how and why RG visitors use RGMA. The questionnaire has been constructed as an exploratory in order to understand the functions and employability of RGMA in the framework of a connected stadium. No pilot has been conducted. Six accredited interviewers have collected the answers for the questionnaires inside the stadium throughout the tournament period. The respondents were selected at random (simple random sampling, Taherdoost, 2016), based on a commitment to take part in this study. All of them are agree that their answers are collected and used in an academic study and published, respecting confidentiality and privacy. Thus, we use the sample of 2018 RG spectators (*N* = 1,000). The event was attended by 480,500 spectators. Also, our sample allows us to have a very good *CI* at 1.85% (with a 95% *CI*). A questionnaire consists of 33 questions regarding spectators' experience on the 2018 RG and socio-demographic variables used for the typology construction. The survey was available in French and English. It is important to note that our sample involves both spectators and accredited persons (volunteers and temporary workers), often younger than 25 years old. We used the results of this Questionnaire to answer research questions, referring to the quantitative method and inductive approach (Tharenou et al., 2007).

### Data Analysis

The questionnaire was analyzed with Modalisa® software^1^. The statistical analysis relies on three types: flat sorting, cross sorting, and factorial correspondence analysis for profile construction based on more than two variables. More precisely, the typology was constructed based on three variables: geographical origin, age category, and involvement in tennis. Correlations in cross-sorting were established using PEM (french abbreviation: pourcentage de l'écart maximum)—percentage from maximum deviation: an index of a tie between modalities of a contingency table (Cibois, 2009).

## Results

The major part of the results, in detail, is disclosed in the Discussion Section, however, in fact, these results make no sense without the authors' interpretation. Results let us answer research questions and achieve the study objective. Notably, the conditions for developing the connected stadium RG are infrastructure and common connectivity (Jäger, 2013), involving personal electronic devices like a smartphone. Deployment of RGMA is one of the conditions of successful development of stadium connectivity. Particularly, 89.7% of spectators notice a benefit from a data connection. However, a few functions have been unsuccessful. In general, the results show that digital tools influence consumer behavior, for example, increasing the probability of switching a court.

### Connected Stadium: Data Connection

At the initial stage, we focused on the infrastructure: first of all, a connected stadium is supposed to offer visitors a high-quality internet connection. The RG stadium is based on a multi-channel technical device to ensure proper coverage of 8.5 ha of the stadium. The data connection of the mobile terminals is first of all provided by a 3G/4G coverage, which relies on dedicated antennas in the two main courts of the stadium. These antennas usually cover the neighborhood, and a complementary device of temporary antennas is set up during the tournament period only. So, the stadium seems to be equipped with all necessary devices for providing a satisfying quality internet connection to visitors.

Nowadays 4G coverage is surpassing Wi-Fi capabilities. Moreover, since the 15th of June 2017 “Roam like at Home” lets within European Union (EU) countries pay domestic prices for roaming calls, SMS, and data. So, we assume “domestic” EU visitors had a mobile internet connection; accordingly, they are not interested in-stadium Wi-Fi.

Otherwise, foreign, non-EU visitors are supposed to be interested in connecting to a Wi-Fi network. From this point of view, the RG stadium does not offer the perfect Wi-Fi connection conditions. There are limited Wi-Fi zones, located outside the courts. The connection is free of charge, but the limits spectators in mobility within the stadium. Under these conditions, people who are limited in their data package or international tourists who are in the roaming zone experienced inconvenience.

### Smartphone Use

The number of smartphone users worldwide is constantly growing^2^ (Statista, 2019, 2021). To answer the questions regarding RGMA, we are also interested in functions mostly used in smartphones in the stadium, including the behavioral changes associated with using RGMA.

### Key Results

To this end, we have outlined the main results:

- The majority (the larger number of the sample) of stadium visitors stay connected
- The majority of visitors are using mobile internet (3G/4G)
- The majority of match spectators use a smartphone during RG stadium visit
- Roland Garros Mobile Application was widely used among RG visitors
- Aged visitors are less flexible in the employment of RGMA
- Tennis fans^3^ download RGMA in advance (before coming to the stadium)
- Tennis fans follow results on the RGMA before and after the tournament
- Following of results of a match *via* RGMA increases the switches of court
- Customers who benefit from a mobile connection (Edge/3G/4G) are more active in content share/posting/publishing
- Tennis fans are the most active group on Social Media in terms of RG.

## Discussion

### General Information

According to the questionnaire results, 89.7% of spectators notice a benefit from a data connection that allows them to download content on their smartphone or to share it around. People who have no connection are significantly more numerous in the age category over 50 years old. Among the “connected” visitors, only 6.8% are dependent on the Wi-Fi network of the stadium, while 82.8% rely on the cellular network (Edge/3G/4G). The most dependent public Wi-Fi hotspots are international tourists^4^. This preliminary analysis of connection possibilities let us know that almost all visitors use their smartphone to interact with devices and applications. In addition, we faced a question: why (for what reasons/tools/functions) did they use smartphones and RGMA while visiting the RG stadium?

It is important to take into account that visits to the RG stadium are often long. The entrance pass (ticket) to the stadium is valid for the whole day. During this period, spectators use their mobile devices for the internet (80.1%), connect to social networks (67.9%) and view/send their emails (66.6%). People aged below 25 years old are significantly more active on social networks, while people over 40 are significantly less active (Table 1).

**Table 1:** Cross-sorting between age category and smartphone usage (blue: negative relation; green: positive relation/dark colors means strong relations).

	**E-mail**	**Internet**	**Social Media**	**RGMA**	**Other**	**Total**
Yonger than 25	127	175	185	94	9	590
From 25 to 40	149	163	149	116	4	581
From 40 to 50	75	91	57	55	5	283
From 50 to 65	70	76	52	57	4	259
65 and older	30	33	20	27	2	112
Total	451	538	463	349	24	1 825

*Khi2 = 29.6, ddl = 16, p = 0.02 (theoritical value <5 = 3) V of Cramer = 0.064*.

Roland Garros Mobile Application 2018 had a late release^5^ on the stores. Nevertheless, 40.8% of people with a data connection downloaded it before coming to the stadium, 2.7% downloaded it on-site and 5.9% still planned to download it. In total, almost half of the responders have RGMA to enrich their experience at the stadium. The range of 25–40 years old is the most actively connected to RGMA. Those who are younger than 25 years old have not always anticipated the download of the application, but they are most likely to install RGMA on-site as soon as they become aware of its existence. The older age categories do not show significant reluctance to the application; however, they are less responsive. If they have not downloaded the application before coming to the stadium, they don't download it on-site by becoming aware of its existence/interest.

Another important aspect refers to the choice to download RGMA: the practice of tennis. There is direct depend: then more people are involved (Raymond, 2016) in tennis (practicing, coming to RG especially for the tennis tournament), the more they use the mobile application.

It was interesting to note that French (domicile) visitors don't use the Wi-Fi connection, probably because they already have a data connection included in their mobile phone subscription. The end of roaming charges in Europe seems to have an effect because, as we can see from collected data, European people typically don't make a special effort to connect to the free Wi-Fi network (except Italians, but our sample includes only three of them). On the contrary, foreign citizens from outside the EU seem to make effort to connect to the stadium's free Wi-Fi network (Table 2). International tourists use RGMA for smart ticketing (Table 3) more actively than French visitors. It may sign about the better awareness of international visitors about the “smart” tool on RG event.

**Table 2:** Crossosrting between domestic countries and connection types (blue: negative relation; green: positive relation/dark colors means strong relations).

	**Pas de connection**	**wifi du stade**	**réseau cellulaire**	**Total**
France	35	14	289	338
UK	3	3	8	14
Belgium	1	1	19	21
Germany	2	4	13	19
Netherlands	3	1	7	11
Spain		1	1	2
Italy		3		3
Extra EU and others:	7	15	55	77
Total	51	42	392	485

*Khi2 = 35.5, ddl = 18, p = 0.008 (Theoritical value <5 = 20) V of Cramer = 0.191*.

**Table 3:** Crossorting between the uses of RGMA and visitors origins (blue: negative relation; green: positive relation/dark colors means strong relations).

	**French**	**International**	**Total**
Planning of event	317	84	401
Following match results	298	75	373
Smart ticketing	106	**53**	159
Stadium plan/navigation	32	11	43
Restaurant reservation	6	2	8
Commercial offers	5		5
Other	25	3	28

*Khi2 = 15.7, ddl = 6, p = 0.015 (Theoritcial val. <5 = 3) V of Cramer = 0.124*.

### Roland-Garros UX With RGMA

The mobile application provides an opportunity to access services that have been synthesized by consulting the schedule of matches, following match results (McCarthya et al., 2013), electronic ticket, stadium/location map, booking a restaurant, benefiting from commercial offers, and receiving other practical information (Ha et al., 2017). The function of informational monitoring of the competition (real-time reports) was one of the main reasons to use the application: the schedule and the results are the main functions exploited by more than 9 users out of 10. An electronic ticket lets the spectators enter the stadium with their smartphones. It was a widely used service (38.5% of people with the application). The use of e-ticketing is the most common reason for the download of the application because a visitor is obliged to install the RGMA on a smartphone/tablet to access the stadium. Additionally, the stadium plan is one of the practical tools that complement on-site spectator support and is used by 10.4% of users.

The lowest scores are related to “additional consumption” (additional, auxiliary, products, and services). Ideally, RGMA could be a tool that helps to increase consumption within the stadium through services (reservations in restaurants) or promotional offers that might trigger shopping behavior (Glebova and Desfontaine, 2020). However, <2% of the users of the application said they were engaged by these functions. Before concluding on the inefficiency of the “additional services consumption” function (Manuika and Roxburgh, 2011; Taylor and Levin, 2014), it is necessary to recall that the late development of the application could contribute to a lower level of integration in the commercial strategies of the service providers in the framework of consolidation all services in a single place. Table 4 demonstrates the usage of RGMA (% on a sub-sample of 413 people who reported downloading the app).

**Table 4:** Usages of Roland Garros Mobile Application (RGMA).

**Items**	**Eff**.	**%**
Matches planning	401	97.1%
Following the results	373	90.3%
Electronical tickets	159	38.5%
Stadium plan/navigation	43	10.4%
Restaurant reservation	8	1.9%
Shopping offers	5	1.2%

We have noticed a strong correlation between using RGMA for following up on matches and the download of the application, especially for tennis fan profiles. Indeed, we observe that a fringe of the public who plays in a club and came to Paris mainly to attend the RG tournament is found to be strongly correlated with the people who downloaded the application in advance (before coming to the RG stadium). We assume that tennis fans follow the results of the tournament on the application before, during, and after they visit the stadium. RGMA may be defined as an accompanying tool for tournament experience that goes beyond the physical space of the stadium, functioning for all the stakeholders (Glebova et al., 2019; Glebova and Desfontaine, 2020).

### Following the Results of the Match on RGMA Increases the Probability of Switching the Court

Inside the stadium, the use of RGMA goes far beyond simply following the results. The connected stadium transforms the customer experience and allows to consume the sports show differently. First of all, the user has easy access to the stadium through the application ticketing service. Moreover, RG is a huge tournament. Matches are played on different courts and scheduling may fluctuate depending on the duration of the matches. The attractiveness of matches can also vary according to both: players' reputations and the convenience of scheduling.

Additionally, RGMA offers the scores and the schedule of matches. Indeed, the spectators constantly follow the evolution of the matches and can change courses according to the attraction of the show. Approximately 64% of RGMA users say that the monitoring (McCarthya et al., 2013) of the competition on their mobile has led them to change courts. We still cannot understand how informational technology determines a consumer behavior in this particular case (Wyatt, 2008). Also, the app helps to improve the customer experience by allowing it to be in the right place at the right time to make the most of your day at the stadium.

### Customers Who Benefit From a Mobile Connection (Edge/3G/4G) Are More Active in Terms of Content Publishing

Customer satisfaction can have a wider beneficial effect. Indeed, each user has an editorial touch that contributes to the global image of the tournament through its publications on the internet and social networks, it's a part of the co-creation process (Horbel et al., 2016). Furthermore, a connected stadium opens up to an expanded, almost uncontrolled communication, corresponding to social constructivism. Unsurprisingly, we have identified a strong correlation between the data connection and the publications on the web. Given the limited number of Wi-Fi hotspots in the RG stadium, customers who benefit from a mobile connection (Edge/3G/4G) are more active in terms of content publishing.

Most of the publications are made on social networks. Direct publication on a blog or website remains unconfirmed. Two main criteria characterize the audiences that are active in this area: age and commitment to practice. Indeed, among our age groups, young people (below 25) and 25–40 years old are active on social networks. For spectators elder than 40 years, the activity of posting/sharing on social media is low.

The third aspect, related to posting activity, is the involvement in tennis communities and willingness to play tennis. People who practice in clubs are significantly more active on social networks. We can assume that they have both reasons to be more active in terms of social posting (1) a community of followers interested in this event and (2) professional interest in a type of publication focused on sports performance and results.

### Typology

A multidimensional analysis of the conducted questionnaire leads us to develop typology and establish profiles of spectators who have different use of RGMA and behavior in the connected stadium of RG (Table 5). Generally, it was based on the following dimensions: age, country of origin, tennis sports affiliation, technology acceptance/technology readiness (Parasuraman and Colby, 2001), perceived RGMA and event benefits (Hautbois et al., 2019), RGMA usage, the intensity of mobile app usage, consumer behavior, content publishing/social media sharing (Table 6).

1. The international tourist represents the large and mixed group of people who come from far away (often outside Europe). Their trip is not centered around the RG and they are not necessarily tennis fans. However, RG is a legendary event and place in Paris, famous throughout the world. So they take advantage of the coincidence between their arrival in Paris and the dates of the tournament to visit the stadium and discover the tournament. Typically, international tourists do not take full advantage of the connected stadium experience. Their connection can be limited and reduced to the Wi-Fi hotspots of the stadium. But the ticket service is an asset that could still lead them to download RGMA. Their variable appeal for tennis will not cause them to constantly connect to the app or actively post on social media.
2. The tennis fan practices the activity regularly in a club or other sports structures. He/she follows the competitions and comes to RG especially for the tournament. To follow the results of the matches, he/she has downloaded RGMA in advance that he uses to track the results and the schedule of the matches. The app will allow him to choose the course on which he will see the matches that interests him the most. Aged between 25 and 40, typically, he is active on social networks and shares his experience with his friends.
3. The youth, young people below 25 of age, are certainly more adjusted and attached to connected devices than elders. They can easily install and use an application a few moments after becoming aware of its existence if they find an interest. They are regular users of social networks and publish regularly. The survey questioned some young people who were not necessarily tennis fans (partly due to the presence of volunteers and temporary workers). This audience can be very active and responsive on the web and social media, supporting a wide range of topics.
4. The seniors are elder than 50 years old, they are numerous among the spectators and many of them practice playing tennis. Among this audience, the experience of the sports show remains traditional in the sense that they are more permeable to digital artifacts. Even if very few do not have a data connection on their smartphone, many of them do not want to download RGMA and prefer other channels to follow the competition. This audience is, therefore, conservative and less receptive to transformations, accordingly, requires more time (Ogburn, 1922; Brinkman and Brinkman, 1997) and effort to be involved in RGMA employment.

**Table 5:** Cross sorting between practicing tennis and usages of RGMA (blue: negative relation; green: positive relation/dark colors means strong relations).

	**Match planning**	**Following results**	**Electronic ticket**	**Plan of stadium/navigation**	**Restaurant reservation**	**Shopping offers**	**Other practical information**
Yes, in club	191	175	66	14	4	4	8
Yes, beyond the club	70	65	37	8	2		5
No	140	133	56	21	2	1	15
Total	401	373	159	43	8	5	28

*Khi2 = 12.5, ddl = 12, p = 0.406 (Theoretical value <5 = 6) V of Cramer = 0.078*.

**Table 6:** Summary of profiles: key differencies.

	**Age**	**Country of origin**	**Tennis sports affiliation**	**TA**	**RGMA usage**	**The intensity of mobile app usage**	**Consumer behavior**	**Perceived benefits**	**Content publishing/social media sharing**
Int. Tourist	The most mixed group in terms of age	From far away, often outside Europe	No affiliation		For ticket purchase only	Low		Visiting legendary place	Low and unrelated to tennis content
Tennis Fan	25–40		Practice tennis in clubs	Medium	Active, Real-time analytics and game schedule	Highest	Following matches before, during, after a visit, changing courts	Follow results	Intensive publishing and sharing tennis-related content with friends and communities
Youth	Below 25			Highest	On purpose, know-how benefit from RGMA tools	Medium		Socialize, explore, work*	High, beyond tennis content
Senior	Over 50		Practice in clubs or belongs to communities	Low	Very low	Low	He doesn't want to download and use RGMA	Follow traditions	Very low

These four profiles of spectators let us think of the stadium connectivity on the users. Also, it encourages sport managers to understand their audience from a new perspective, and, subsequently, to suggest complementary strategies in order to improve customer experience in the RG stadium, and other stadia as well.

### Conclusions

Analyzing literature, context, and empirical data let us understand differences between RG event visitors in terms of their employment and use of RGMA. Finally, we have distinguished 4 main types of RGMA users: (1) international tourist, (2) tennis fan, (3) youth, and (4) senior.

Age is a crucial characteristic in terms of using an app. Young people easier accept, maintain, and use new digital technologies and know-how to benefit from that. On average, visitors over 50 are less flexible in the employment of mobile apps and rarely interested in using them.

No doubt, the quality of available internet and continued connectivity occurring all the time are the key conditions for creating a connected stadium. However, the majority of stadium visitors uses their smartphones and keep staying connected on mobile data packages, neglecting stadium Wi-Fi.

Anyway, the majority of visitors have been using RGMA and then more a visitor is involved in practicing tennis, then more active he/she is in using RGMA. Tennis fans follow the results of the tournament on RGMA before and after their visit to the stadium, downloading the app in advance, before coming to the stadium. They are more active in terms of content publishing and social media share, especially in the age below 40.

Overall, the main advantage and convenience of the mobile app is a consolidation of all services and information in a single place. It's comfortable and practical for all stakeholders in terms of customer relation management (CRM) and data collection. The smart ticketing option is far from new but highly appreciated by customers, sometimes being a driver for app download. Real-time analytics is mainly used by tennis fans and increases the probability of a spectator's decision to change court during the event.

### Limitations

We used a simple random sample (*N* = 1,000) within a large group of RG 2018 visitors (*N* = 480,500), and we believe that this sample is sufficient to represent the targeted population in an unbiased way and answer research questions (Taherdoost, 2016). However, the representation of all visitors of the event is skewed and requires additional sampling techniques. Also, the data collection method involves a structured questionnaire; the respondents had limited options of responses, based on the questionnaire content. It may lead to quite limited outcomes, missing further “how” and “why” (qualitative perspective/approach) to describe and explain phenomena. In this regard, over the paper, we let ourselves add our own authors' observations to provide more information and explain unclear actual occurring.

### Future Research Directions

The framework of IoT and internet coverage technology encourages fans to buy tickets, find their seats, check toilets' availability, find parking, and order food, ultimately transforming the regular match day experience. Immersive technologies such as augmented reality are also being implemented enabling fans to see additional information such as exclusive footage, survey stats, and analytics. Brand image, brand attitude, brand awareness, and brand focus thereby brand equity and value can be affected with a mobile application (Vikström and Zheng, 2013). The rapid development and diffusion of various technological tools in sports spectacle raises many questions for scholars, especially from the consumer-centric perspective.

## Data Availability Statement

The raw data supporting the conclusions of this article will be made available by the authors, without undue reservation.

## Ethics Statement

Ethical review and approval was not required for the study on human participants in accordance with the local legislation and institutional requirements. Written informed consent for participation was not required for this study in accordance with the national legislation and the institutional requirements.

## Author Contributions

P-OS and EG contributed to the conception and design of the study and together wrote the first draft of the manuscript. P-OS collected and organized the database and performed the statistical analysis. EG wrote sections of the manuscript. All authors contributed to manuscript revision, read, and approved the submitted version.

## Funding

This work was partially supported by the ADI 2018 project, funded by IDEX Paris-Saclay, ANR-11- IDEX-0003-02.

## Conflict of Interest

The authors declare that the research was conducted in the absence of any commercial or financial relationships that could be construed as a potential conflict of interest.

## Publisher's Note

All claims expressed in this article are solely those of the authors and do not necessarily represent those of their affiliated organizations, or those of the publisher, the editors and the reviewers. Any product that may be evaluated in this article, or claim that may be made by its manufacturer, is not guaranteed or endorsed by the publisher.

## Footnotes

^1^ https://www.modalisa.com/

^2^https://www.statista.com/statistics/330695/number-of-smartphone-users-worldwide/; https://www.statista.com/statistics/330695/number-of-smartphone-users-worldwide/#:~:text=Smartphone%20users%20worldwide%202016%2D2021&text=The%20number%20of%20smartphone%20subscriptions,in%20the%20next%20few%20years

^3^Typology of current paper, please see subsection 4.5.

^4^Typology of current paper, please see subsection 4.5.

^5^RGMA was available on April 17 but several functions were available with updates (6 between May 18 and June 6) source: https://apps.apple.com/fr/app/roland-garros-officiel/id137158479.

## References

[B1] Behrens A. Uhrich S. (2021). You'll never want to watch alone: the effect of displaying in-stadium social atmospherics on media consumers' responses to new sport leagues across different types of media. Europ. Sport Manage. Quart. 10.1080/16184742.2021.1992469

[B2] Brinkman R. L. Brinkman J. E. (1997). Cultural lag: conception and theory. Int. J. Soc. Econ. 24, 609–627. 10.1108/0306829971017902618264028

[B3] Buck C. Horbel C. Germelmann C. C. Eymann T. (2014). “The unconscious app consumer: discovering and comparing the information-seeking patterns among mobile applications consumers,” in Twenty-Second European Conference on Information Systems, Tel Aviv 2014.

[B4] Cibois P. (2009). Percentage of Maximum Deviation From Independence (PEM): Comment on Lefèvre & Champely's “Analyse D'un Tableau de Contingence” Article. Bull. Soc. Methodol. 103, 66–74. 10.1177/075910630910300107

[B5] Glebova E. Desbordes M. (2021). Technology innovations in sports: typology, nature, courses and impact, Chapters, in Innovation and Entrepreneurship in Sport Management, chapter 5, ed Ratten V. (Edward Elgar Publishing), 57–72. 10.4337/9781783473960.00012

[B6] Glebova E. Desbordes M. Geczi G. (2019). Changes in-stadia sports spectators customer experiences. Physical Education, Sport, Science (PSS), Testnevelés, Sport,Tudomány (TST). TST/PSS 2019, 65–75. 10.21846/TST.2019.3-4.6

[B7] Glebova E. Desfontaine P. (2020). Sport et technologies numériques: vers de nouvelles expériences spectateur (Sport and digital technologies: towards new spectator experiences), in Management du sport 3.0, Spectacle, Fan Experience et Digital. Economica, eds Desbordes M. Hautbois C. (Paris: Economica), 245–270.

[B8] Greenhalgh G. Dwyer B. Biggio B. (2014). There's an app for that: the development of an NFL team mobile application. J. Appl. Sport Manage. 6.

[B9] Ha J.-P. Kang S. J. Kim Y. (2017). Sport fans in a “smart sport” (SS) age: drivers of smartphone use for sport consumption. Int. J. Sports Market. Sponsorship 18, 281–297. 10.1108/IJSMS-08-2017-093

[B10] Hautbois C. Djaballah M. Desbordes M. (2019). The social impact of participative sporting events: a cluster analysis of marathon participants based on perceived benefits. Sport Soc. 23, 335–353. 10.1080/17430437.2019.1673371

[B11] Horbel C. Buck C. Diel S. Reith R. Walter Y. (2021). Stadium visitors' smartphone usage and digital resource integration. Sport Business Manage. 11, 10–27. 10.1108/SBM-10-2019-0099

[B12] Horbel C. Woratschek H. Popp B. (2016). Value co-creation, in Handbuch Dienstleistungsmanagement, eds Corsten H. Roth S. (München: Vahlen), 63–78.

[B13] Hwang H. Yang H. Williams A. Pedersen P. (2020). A gratification model of sport team mobile application usage. Sport Market. Quart. 29, 163–176. 10.32731/SMQ.293.092020.01

[B14] Jäger M. (2013). The Advent of the Fan Experience 2.0 Through Wi-Fi? The Connected Stadium. Research Report. Sport Facility Management.

[B15] Kang S. J. Ha J. Hambrick M. E. (2015). A mixed-method approach to exploring the motives of sport-related mobile applications among college students. J. Sport Manage. 29, 272–290. 10.1123/jsm.2013-0065

[B16] Kemppainen J. (2018). Designing user experience of an application for live ice hockey game context. [dissertation/master's thesis]. The Tampere University of Technology.

[B17] Manuika J. Roxburgh C. (2011). The Great Transformer: The Impact of the Internet on Economic Growth and Prosperity. McKinsey Global Institute.

[B18] McCarthya M. W. Jamesa D. A. Rowland D. D. (2013). Smartphones: Feasibility for real-time sports monitoring, 6th Asia-Pacific Congress on Sports Technology (APCST). 10.1016/j.proeng.2013.07.044

[B19] McKinley J. (2015). Critical argument and writer identity: social constructivism as a theoretical framework for EFL academic writing. Critic. Inquiry Lang. Stud. 12, 184–207. 10.1080/15427587.2015.1060558

[B20] Næss H. E. (2016). Spectator experience management at FIA World Rally Championship events. Sci. Forum Sport Manage. 12, 49–60. 10.4127/ch.2016.0112

[B21] Ogburn W. (1922). Social Change With respect to Nature and Original Cult. New York, NY: Viking.

[B22] Parasuraman A. Colby C. (2001). Techno-Ready Marketing: How and Why Your Customers Adopt Technology. New York, NY: The Free Press.

[B23] Raymond N. A. (2016). Examining Motivation to Participate in Sport: A Retrospective Look at Current and Former Athletes' Motivation to Participate in Athletics” (2016). Theses and Dissertations (All). 1398. Available online at: https://knowledge.library.iup.edu/etd/1398 (accessed October 20, 2021).

[B24] Taherdoost H. (2016). Sampling methods in research methodology; how to choose a sampling technique for research. Int. J. Acad. Res. Manage. 5.

[B25] Taylor D. G. Levin M. (2014). Predicting mobile app usage for purchasing and information sharing. Int. J. Retail Distrib. Manag. 42, 759–774. 10.1108/IJRDM-11-2012-0108

[B26] Tharenou P. Donohue R. Cooper B. (2007). Management Research Methods. Cambridge University Press. 10.1017/CBO9780511810527

[B27] Veal A. J. Darcy S. (2014). Research Methods in Sport Studies a Sport Management, A Practical Guide. London: Routledge. 10.4324/9781315776668

[B28] Vikström H. Zheng C. (2013). Branding through mobile applications – A case study of Swedish campaign applications, [dissertation/master's thesis] INDEK.

[B29] Winner L. (2001). Where technological determinism went, in Visions of STS: Counterpoints in Science, Technology and Society Studies, 11–17.

[B30] Wyatt S. (2008). Technological determinism is dead; long live technological determinism, in The Handbook of Science and Technology Studies, Vol. 3, ed Wyatt S. (MIT Press) 165–180.

